# Development of the MetFlex Index™: associations between cardiometabolic risk factors and fitness using a novel approach with blood lactate

**DOI:** 10.3389/fphys.2025.1546458

**Published:** 2025-04-14

**Authors:** Bryan Jasker, Daniel Dodd, Clara B. Peek, Garett J. Griffith

**Affiliations:** ^1^ Northwestern University Department of Physical Therapy and Human Movement Sciences, Chicago, IL, United States; ^2^ OVAL, Greenwood Village, Denver, CO, United States; ^3^ Illinois Wesleyan University School of Nursing, Bloomington, IL, United States; ^4^ Northwestern University Feinberg School of Medicine Department of Biochemistry and Molecular Genetics, Chicago, IL, United States; ^5^ Division of Endocrinology, Northwestern University Feinberg School of Medicine Department Medicine, Metabolism and Molecular Medicine, Chicago, IL, United States

**Keywords:** cardiometabolic fitness, lactate, metabolic flexibility, mitochondria, metabolism

## Abstract

**Introduction:**

Cardiometabolic health is declining in the U.S. and anticipated to worsen over the next 30 years. Measurements of cardiometabolic health include blood metabolite profiles. One such metabolite is blood lactate. Lactate assessment is common in critical care and performance settings but less frequently used for the general population. The delayed onset of lactate accumulation during exercise may be an indicator of cardiometabolic health. Assessing lactate during a submaximal exercise test may assist in describing cardiometabolic health status in terms of metabolic fitness and metabolic flexibility.

**Objectives:**

To introduce the MetFlex Index™ (MFI), a novel, scalable exercise-based and marker of cardiometabolic health, and to characterize its associations with routinely assessed cardiometabolic health risk factors.

**Methods:**

Participants completed a submaximal test on a commercial stationary cycle following assessments of body composition, anthropometrics, vital signs, and a blood draw. Lactate was collected at each stage and the 1^st^ and 2^nd^ lactate thresholds were described. The MFI was calculated by using the power, in Watts, attained at the 1^st^ lactate threshold relative to the participant’s Body Mass Index (BMI).

**Results:**

Data were collected on 827 participants (43 ± 13 years, 67% male, 72% overweight or obese). MFI peaked in the 30–39 year old cohort and decreased in subsequent decades. MFI was negatively associated with most markers of anthropometry, body composition, blood pressure, and was not associated with most blood metabolites.

**Discussion:**

The MetFlex Index™ is a novel exercise-based approach using blood lactate to characterize skeletal muscle metabolism and is associated with several cardiometabolic health indices.

## 1 Introduction

Cardiometabolic health is in decline. Prevalence data demonstrate that 40% of U.S. adults are living with obesity (Hales et al., 2020) and 15% of adults are living with diabetes (CDC Diabetes Statistics Report) which may continue to increase over the next 30 years (Maddox et al., 2024). Physical inactivity and sedentary behavior contribute to increases in the severity of metabolic risk factors (i.e., Metabolic Syndrome) which include high blood glucose, low high-density lipoprotein (HDL), high triglycerides, high blood pressure, and large waist circumference (Booth et al., 2017; Rudwill et al., 2018; Rynders et al., 2018; Cunningham et al., 2020; Pillon et al., 2020; Tabozzi et al., 2020; Roux et al., 2022). Yet, physical activity and exercise can be effective attenuators of metabolic disease and contribute to improved tissue and organ function beyond skeletal muscle (Thyfault and Bergouignan, 2020) and this can be independent of weight loss. The effects of activity or inactivity are quantifiable through changes in cardiorespiratory fitness (CRF). CRF positively modifies the risks related to metabolic dysfunction (Ross et al., 2016), including those living with an elevated BMI (Khan et al., 2021).

Cardiopulmonary exercise testing (CPET) is a method to measure gas exchange and to directly quantify maximum oxygen utilization (VO_2max_). Another method that has been useful for measuring CRF and submaximal markers of fitness is blood lactate testing. Historically, lactate had been described as a waste product associated with hypoxia and fatigue (Ferguson et al., 2018). More recently, however, lactate has undergone a rebranding as the “fulcrum of metabolism” (Brooks, 2020), demonstrating significance as a systemic biomarker in metabolism and as a substrate for mitochondrial metabolism and respiration (Broskey et al., 2020; Glancy et al., 2021). Although lactate is an established metabolic biomarker in critical care settings and human performance, lactate remains underdeveloped as a metabolic marker of health and disease (Poole et al., 2021), particularly in the general population. Some developments include lactate as a metabolic biomarker based on associations with resting and fasting plasma lactate levels (Jones et al., 2019; Broskey et al., 2021) and describing metabolic flexibility with substrate oxidation relative to lactate clearance during a graded exercise test (GXT) (San-Millán and Brooks, 2018).

Gaps also persist in understanding the relationships between the onset and development of metabolic dysfunction relative to metabolic flexibility and fitness, as shown in the association between metabolic inflexibility, low CRF, and ectopic lipid accumulation (Galgani and Fernández-Verdejo, 2021). Developing additional methods comprising active assessments to explore differences in internal and external loads (stress) may prove useful to describe the relationships between metabolic health and fitness more effectively. To address these gaps, it is important to develop less invasive, scalable, and movement-based approaches for quantifying metabolic flexibility (Rynders et al., 2018) relative to CRF to assess the efficacy of various interventions within clinical and nonclinical populations.

Herein, we describe a novel scoring system using blood lactate as an indicator of metabolic health and fitness, complementing traditional methods for describing CRF. The feasibility of scaling this novel marker of skeletal muscle metabolism in the general population is discussed. The primary purpose of this study was to define and quantify a marker of metabolic response to exercise, the MetFlex Index™, in healthy adults. Secondarily, we aimed to characterize associations of the MetFlex Index™ with routinely assessed health indicators and cardiometabolic health risk factors.

## 2 Materials and methods

### 2.1 Study population

Eight hundred and twenty-seven (n = 827) healthy adults from various populations in the U.S. and Canada were included in this study. Participants included: first responders (police and fire), active and retired military personnel, city government office-based employees, fitness center/health club members, and individuals from lab-based and mobile corporate wellness settings. Three hundred and ninety-six (48%) of the participants were part of employee-based general health and wellness programs that included metabolic fitness testing (GXT) with their biometric assessments or were retail customers of a general wellness program. Participants were excluded if they had any conditions or limitations preventing the ability to participate in a submaximal GXT on an upright or recumbent cycle, if a physician recommended exercise only under the supervision of a medical professional, or if a participant reported fever, malaise, or illness within 3 days prior to testing. Testing data were collected from 10 October 2020, to 1 July 2024. Testing took place over 4 years in various community health and wellness testing programs where not all testing variables were available for sampling, including bioimpedance and point-of-care blood metabolites, leading to differing sample sizes relative to the entire cohort that tested. All participants provided written informed consent as an “opt-in” prior to any testing. They received instructions for test preparation at least 48 h prior to their testing date. Per preference, participants either registered on an app they downloaded to their smartphone, or they registered directly with a tester. Data entered during registration included name, birthdate, sex, cell number, email, city, and zip code. Protocols for the GXT resided on a testing application based within a tablet. All health screening and testing data were de-identified prior to this analysis. Only data from each participant’s initial GXT were included. This study received IRB approval to conduct secondary analyses of the previously collected data.

### 2.2 Body composition and anthropometrics

Body composition was assessed via bioelectric impedance analysis (InBody 770 bioimpedance, InBody USA, California, USA). Bioimpedance testing was limited to a subset of participants due to limited resource availability in some community settings. An overnight fast was recommended prior to the scan, although not all participants could comply if testing were not in the AM. In the case of an afternoon test, participants ate breakfast but withheld lunch or snacking. Voiding, bowel movements, and avoiding excessive rehydration were encouraged prior to the scan. A single scan of each participant was recorded, and a physical copy of the report was printed and provided to each participant. Waist circumference was measured at the level of the naval in a standing position. The participant held the end of the tape on the naval with a fingertip and then turned one full rotation, maintaining parallel position to the floor, and returning to the starting position. Measuring directly on the individual’s skin was preferred, however, per preference, circumference was also measured over clothing if the clothing was thin as in athletic attire. A single measurement was taken in a relaxed position and recorded in the nearest half centimeter.

### 2.3 Vital sign assessment

Vital signs were measured prior the submaximal GXT consistent with ACSM participation guidelines, 11th edition. All participants received a single reading of resting heart rate (HR), resting blood pressure (BP), and resting pulse oximetry (PO). These measures were taken after 3 minutes of resting in a seated position in a chair with back support and feet flat on the floor. Resting HR and PO were measured from the same device (OxyWatch C20, ChoiceMed, Illinois, USA). After appropriate cuff sizing, blood pressures were taken manually when available otherwise an automatic cuff was used (Omron, Japan). No vital signs were actively monitored during the submaximal GXT except for heart rate.

Vital sign analysis also included double product (rate pressure product) and chronotropic index. Double product is the result of resting systolic BP multiplied by resting HR and has been associated with cardiac work and myocardial oxygen consumption (Gobel et al., 2018). Chronotropic index is used to assess the heart’s ability to increase its rate relative to an increase in physical stress and metabolic demand. It provides insight into autonomic nervous system function and cardiovascular risk (Sirichana et al., 2020). The chronotropic index is calculated by dividing the difference between the measured peak HR and the resting HR by the difference between the age-predicted maximal HR and the resting HR.

### 2.4 Point-of care blood metabolite profiling

Some participants received wellness-based, point-of-care blood sampling for HbA1C (A1CNow Self Check, PTS Diagnostics, Indiana, USA), fasting glucose (Mentene 4,116, China), fasting lactate (Nova Biomedical, Boston, USA), and lipid profiling (Curo L7, California, USA). Point of care testing was limited to a subset of participants due to limitations of resource availability in community settings. Participants were instructed to arrive in an overnight or morning fast (given morning or afternoon testing) and normally hydrated. All capillary blood access was from a single fingerstick using a 23G safety spring lancet.

### 2.5 Submaximal, graded exercise test

**FIGURE 1:**
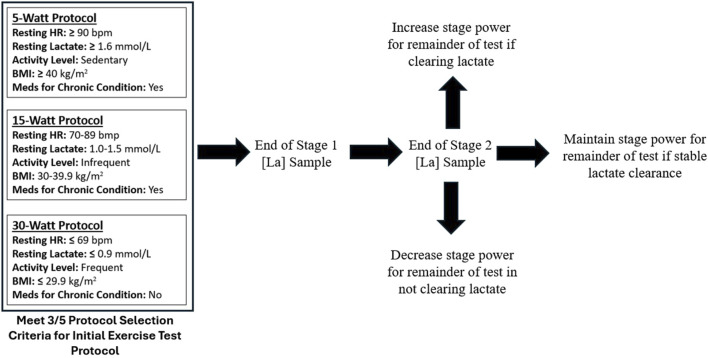
Adaptive exercise testing protocols.

All data including anthropometrics, vital signs, and blood metabolites were digitally entered into a metabolic testing platform application (OVAL). Submaximal GXT protocols were performed with concurrent measurement of blood lactate. The GXT was performed on various commercial grade upright cycles with capacity to monitor resistance (Watts) and revolutions per minute (RPMs). Cycling was chosen for safety, power (Watts), and convenience of portability for testing. Participants started the GXT in one of three protocols determined by five variables (Protocol Selection Criteria): resting HR, resting lactate, current activity level, BMI, and medications for chronic condition (Figure 1.). Protocols were adaptable given the participant’s ability to clear or accumulate lactate during the first two stages of the exercise test. If a participant was able to clear (reduce) lactate levels, the Watts per stage could increase or remain the same. If a participant was unable to clear lactate or demonstrated early accumulation, then the Watts per stage could decrease or remain the same.

### 2.6 Lactate analysis

Lactate was collected from capillary blood and analyzed with a commercial-based lactate meter intended for sports and performance applications (Lactate Plus, Nova Biomedical, Waltham, MA). Capillary blood was accessed from the bottom of an earlobe using a 23G safety spring lancet. The blood lactate sampling cadence at this site was at the end of each 3-min stage of the GXT. Algorithms identifying the 1^st^ and 2^nd^ lactate thresholds within each protocol remain proprietary (OVAL). Generally speaking, the 1^st^ lactate threshold was defined as a specific lactate concentration which was relative to the protocol completed (either 5, 15, or 30 W). Meaning the lower the protocol completed (5 W) then the greater the lactate concentration defined for LT1 and the higher the protocol completed (30 W) then the lower the threshold concentration defined for LT1. If the defined lactate level was not obtained or in-between values, then interpolation was used. The 2^nd^ lactate threshold was defined as a specific lactate concentration regardless of protocol. Methods of interpolation were used by comparing two stages. Submaximal GXT was complete when 1) blood lactate reached at least 5.9 mmol, 2) the participant voluntarily ceased the testing, or 3) the technician supervising the GXT ceased testing. Peak lactate of 5.9 mmol was selected as 1) this is a submaximal effort test, 2) this level is likely above a second lactate threshold for most participants, and 3) participants are typically demonstrating an increased rate of lactate accumulation by this point given their current level of fitness.

### 2.7 MetFlex Index™

The MetFlex Index™ was developed to aid in the measurement and management of metabolic health and fitness. The MetFlex Index™ is a power to mass ratio like the W/kg metric in cycling with two differences: 1) the MetFlex Index™ identifies the power attained at the first lactate threshold rather than the second lactate threshold and 2) the power in Watts at the first lactate threshold is relative to BMI instead of an individual’s mass in kilograms. The MetFlex Index™ is a continuous scale from 0–100+ (Supplementary Figure 1) where lower scores are characteristic of metabolic inflexibility, lower oxidative capacity, and greater reliance on glycolysis during daily activities. Higher MetFlex Index™ scores are more characteristic of metabolic flexibility, higher oxidative capacity, and less reliance on glycolysis during regular daily activities. For the purposes of describing differences between lower and higher scores, we grouped participants into five categories based on their MetFlex Index™: 0–9.9, 10–19.9, 20–29.9, 30–39.9, and 40+. Each MetFlex Index™ category and its relative mean VO_2_ and MET value are shown in Table 1.

**TABLE 1:** Relative VO2 and METs.

MetFlex index	Mean VO2(SD)	Mean METs (SD)
0–9.9	5.6 (1.3)	1.6 (0.4)
10–19.9	9.5 (1.1)	2.6 (0.3)
20–29.9	12.4 (1.3)	3.5 (0.4)
30–39.9	15.5 (1.4)	4.4 (0.4)
40+	22.0 (5.3)	6.3 (1.5)

### 2.8 Statistical analysis

Continuous variables were analyzed as mean (standard deviation). Associations between the MetFlex Index™ and known cardiometabolic health indicators were investigated using bivariate correlations and Pearson correlation coefficients. Differences in cardiometabolic health indicators based on MetFlex Index™ groups were identified using analyses of variance (ANOVA), with Bonferroni *post hoc* testing to confirm the nature of significant differences between MFI categories. Statistical significance for all analyses was set at p < 0.05 and all statistical analyses were performed using opensource software including pandas, NumPy, statsmodels, or SciPy for statistics and matplotlib and seaborn for graphs.

## 3 Results

This was a multi-site, multi-population observational study conducted over approximately 45 months. All participants performed a GXT including anthropometrics and vital sign assessment. Analysis of body composition (bioimpedance) and blood metabolites were also performed when available.

### 3.1 Participant characteristics

A total of 827 participants were enrolled into this study. Sociodemographic, anthropometric, resting vital signs, and blood metabolite data for study participants are in Table 2. Participants were aged 43 ± 13 years and 67% were male. Over half of the study samples were overweight (*n* = 351, 42.5%) or obese (*n* = 247, 29.9%). Advanced body composition analysis was available in 178 participants and fasting metabolic blood metabolite data were collected in 39 participants.

**TABLE 2:** Age, sex, and MetFlex index.

Age	MetFlex index (SD)	Sample size (n)
18–29	29.8 (16.8)	119
30–39	33.6 (18.9)	218
40–49	32.6 (19.0)	239
50–59	28.7 (18.1)	147
60–69	25.6 (16.8)	66
70–79	21.2 (13.2)	17
80+	15.6 (10.1)	5
Under 65	31.5 (18.5)	766
65+	20.5 (13.1)	52

Sex	MetFlex Index (SD)	Sample size (n)
Male	33.3 (18.5)	557
Female	25.7 (16.9)	270
Total	30.8 (18.3)	827

### 3.2 MetFlex Index™: age and sex

The distribution of MetFlex Index™ scores across ages 18–80+ years is shown in Table 3. MetFlex Index™ was 29.8 ± 16.8 in those aged 18–29 years, peaked in those aged 30–39 years (33.6 ± 18.9), and decreased during each subsequent decade of life thereafter. Those aged ≥80 years demonstrated the lowest MetFlex Index™ (15.6 ± 10.1). Mean values of the MetFlex Index™ for males and females are shown at the bottom of Table 3. Males recorded a higher MetFlex Index™ than females (7.6 points, *p* = 0.001).

**TABLE 3:** Participant characteristics.

Variables	Mean (SD)	Sample size (n)
Age (yr)	43 (13)	818
Male (%)	67%	557
MetFlex Index	30.8 (18.3)	826
Body Mass Index (kg/m2)	28.4 (5.7)	826
Body Mass Index category		
<24.9	22.6 (1.7)	228
25–29.9	27.3 (1.4)	351
30+	35.2 (5.2)	247
Waist Circumference (cm)	95.0 (15.5)	816
Body Fat Mass (lbs)	59.7 (30.3)	178
Percent Body Fat (%)	29.4 (9.9)	178
Visceral Fat Area (cm^2^)	128.8 (62.3)	168
Lean Body Mass (lbs)	139.1 (32.4)	176
Skeletal Muscle Mass (lbs)	78.7 (19.6)	178
Percent Skeletal Muscle (%)	39.7 (6.0)	178
Resting heart rate (bpm)	71.7 (11.8)	826
Systolic blood pressure (mmHg)	129.8 (14.5)	826
Diastolic heart rate (mmHg)	81.3 (9.4)	826
Mean Arterial Pressure (mmHg)	97.5 (9.9)	826
Mean Pressure Product	9,327.3 (1931.7)	826
Chronotropic index	0.85 (0.17)	666
Fasting blood lactate (mmol)	1.8 (0.8)	39
Resting blood lactate (mmol)	1.2 (0.4)	826
Fasting blood glucose (mg/dL)	105.0 (26.0)	39
Triglycerides (mg/dL)	107.6 (54.6)	38
Total cholesterol (mg/dL)	160.8 (46.3)	39
HbA1C (%)	5.7 (1.2)	38

### 3.3 MetFlex Index™ and anthropometrics (body composition)

**FIGURE 2:**
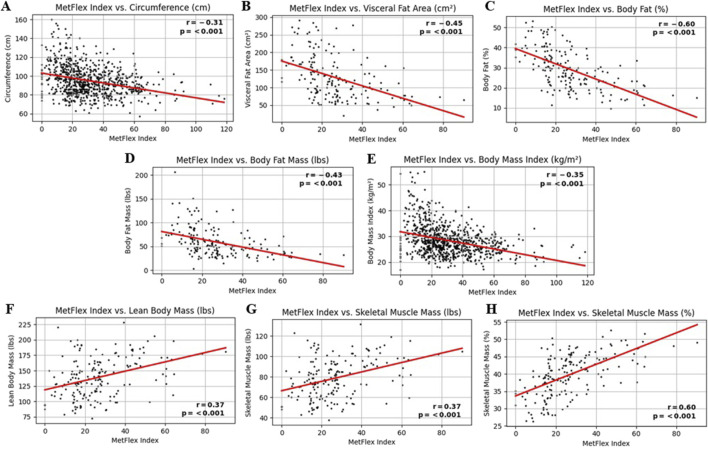
**(A-H)** MetFlex Index^™^ and anthropometrics.

The MetFlex Index™ was significantly and negatively correlated with several markers of anthropometry and body composition, including, waist circumference (*r* = −0.31, *p* < 0.001), visceral fat area (*r* = −0.45, *p* < 0.001), percent body fat (*r* = −0.60, *p* < 0.001), body fat mass (*r* = −0.43, *p* < 0.001), and body mass index (*r* = −0.35, *p* < 0.001). The MetFlex Index™ was significantly and positively correlated with lean body mass (r = 0.37, *p* < 0.001), skeletal muscle mass (r = 0.37, *p* < 0.001), and percent skeletal muscle mass (r = 0.60, *p* < 0.001). These associations are shown in Figures 2A–H.

### 3.4 MetFlex Index™ and vital signs

**FIGURE 3:**
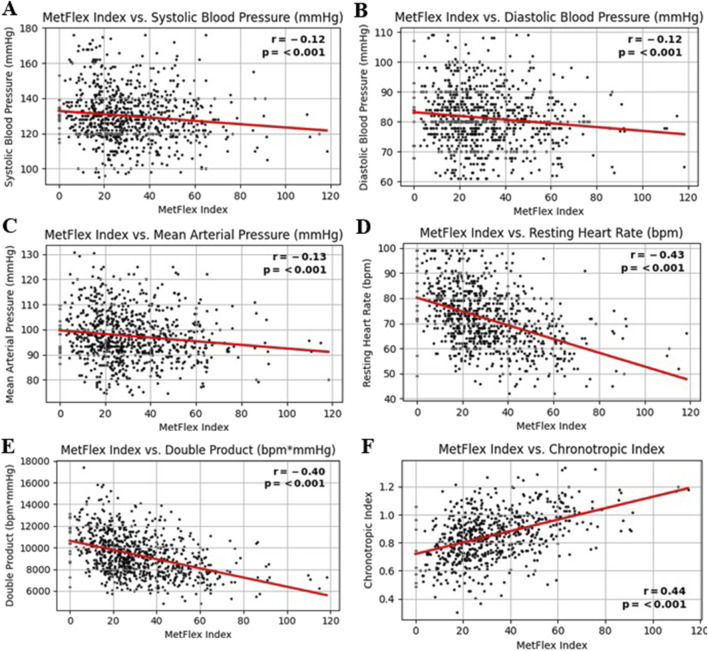
**(A-F)** MetFlex Index^™^ and vital signs.

The MetFlex Index™ was significantly and negatively correlated with cardiovascular metrics including systolic blood pressure (*r* = −0.12, *p* < 0.001), diastolic blood pressure (*r* = −0.12, *p* < 0.001), mean arterial pressure (*r* = −0.13, *p* < 0.001), resting heart rate (*r* = −0.43, *p* < 0.001), and (resting) double product (*r* = −0.43, *p* < 0.001). The MetFlex Index™ was significantly and positively correlated with the chronotropic index (*r* = 0.44, *p* < 0.001). These associations are shown in Figures 3A–F.

### 3.5 MetFlex Index™ and blood metabolites

**FIGURE 4:**
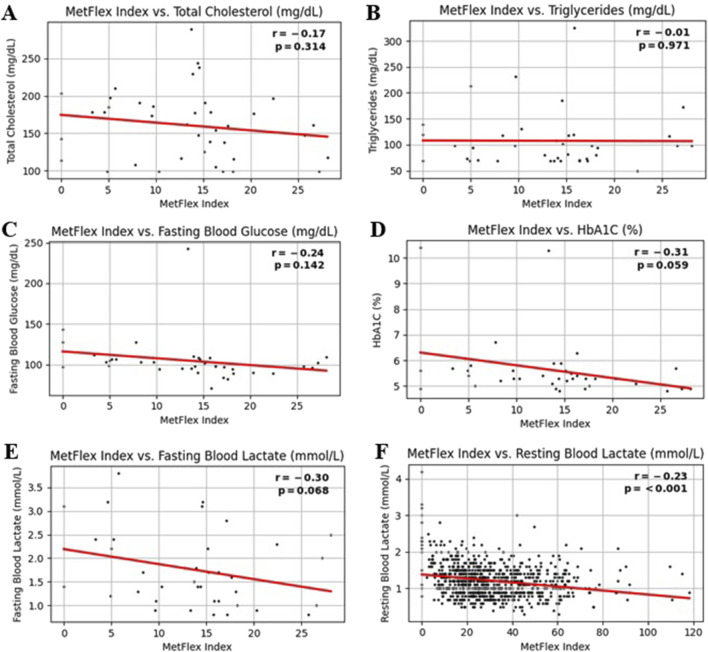
**(A-F)** MetFlex Index^™^ and blood metabolites.

The MetFlex Index™ showed no significant correlation with most blood metabolites, including total cholesterol (*r* = −0.17, *p* = 0.314), triglycerides (*r* = −0.01, *p* = 0.971), fasting blood glucose (*r* = −0.24, *p* = 0.142), or HbA1C (*r* = −0.31, *p* = 0.059), fasting blood lactate (*r* = −0.30, *p* = 0.068). Resting blood lactate (*r* = −0.23, *p* = 0.001) demonstrated a significant and negative correlation. This data is shown in Figures 4A–F. These data are from a sample size of n = 39 and therefore should be viewed as preliminary.

There were differences in anthropometric and body composition outcomes according to MetFlex Index™ categories (Table 4), including BMI (*p* < 0.001), waist circumference (*p* < 0.001), percent body fat (*p* < 0.001), and visceral fat area (*p* < 0.001). A pattern emerged where each MetFlex Index™ category, observed from lowest (0–9.9) to highest (40+), scaled inversely with most cardiovascular health indicators (Table 4). Interestingly, there were limited observations of dysfunctional cardiometabolic health in the 40+ MetFlex Index™ category which may imply a minimal metabolic fitness threshold for sustaining health. No significant correlations were associated with most blood metabolite profiles.

**TABLE 4:** MetFlex Index^™^ groups and metabolic-related variables.

*Secondary Variables*	MFI 0 to 9	MFI 10 to 19	MFI 20–29	MFI 30–39	MFI 40+
Body mass index (kg/m^2^)	32.8 (8.6) ^^*#$^	30.2 (6.4) ^!*#$^	27.7 (4.5) ^!^^^$^	27.0 (4.1) ^!^^^$^	26.1 (3.9) ^!^^^*#^
Waist circumference (cm)	104.9 (19.2) ^^*#$^	99.3 (19.5) ^!*#$^	93.1 (13.1) ^!^^^$^	91.7 (12.7) ^!^^	89.6 (12.3) ^!^^^*^
Percent body fat (%)	41.2 (7.2) ^^*#$^	33.9 (9.4) ^!*#$^	27.8 (7.7) ^!^^^$^	26.2 (6.7) ^!^^^$^	20.2 (6.0) ^!^^^*#^
Visceral adipose Tissue (cm^2^)	192.5 (55.2) ^^*#$^	149.6 (64.5) ^!*#$^	115.7 (50.7) ^!^^^$^	107.1 (60.6) ^!^^	90.9 (36.8) ^!^^^*^
Percent Skeletal Muscle (%)	32.9 (4.4) ^^*#$^	37.1 (5.7) ^!*#$^	40.6 (4.7) ^!^^^$^	41.8 (4.1) ^!^^^$^	45.2 (4.2) ^!^^^*#^
Systolic blood pressure (mmHg)	132.9 (14.0)^*#$^	131.6 (15.8)^*$^	127.2 (13.2) ^!^^	129.0 (13.3) ^!^	128.5 (15.5) ^!^^
Diastolic blood pressure (mmHg)	83.3 (9.7)^*$^	81.8 (9.3) ^$^	80.3 (9.1) ^!^	81.5 (9.2)	80.0 (9.7) ^!^^
Resting Heart Rate (bpm)	82.1 (11.1) ^^*#$^	76.3 (11.2) ^!*#$^	73.3 (10.7) ^!^^^$^	71.5 (10.8) ^!^^^$^	65.3 (11.6) ^!^^^*#^
Resting blood lactate (mmol)	1.7 (0.7) ^^*#$^	1.3 (0.4) ^!*#$^	1.2 (0.3) ^!^^^#^	1.1 (0.3) ^!^^^*^	1.1 (0.4) ^!^^
Mean arterial pressure (mmHg)	99.9 (9.9)^*$^	98.4 (10.3)^*$^	95.9 (9.4) ^!^^	97.3 (9.7)	96.2 (10.5) ^!^^
Pulse pressure (mmHg)	49.6 (11.7)	49.8 (12.5)^*^	46.9 (10.7) ^	47.5 (9.8)	48.5 (12.2)
Double Product (bpm*mmHg)	10,906.7 (1841.5) ^^*#$^	10,056.2 (1953.8) ^!*#$^	9,318.9 (1,623.2) ^!^^^$^	9,218.4 (1,680.1) ^!^^^$^	8,369.0 (1741.4) ^!^^^*#^
Fasting blood glucose (mg/dL)	111.0 (13.6)^*^	103.3 (34.3)	97.3 (7.5) ^!^		
Fasting blood lactate (mmol)	2.1 (0.9)	1.6 (0.7)	1.6 (0.8)		
Total cholesterol (mg/dL)	166.7 (37.8)	160.4 (54.5)	149.8 (36.4)		
HbA1C (%)	5.9 (1.4)	5.6 (1.2)	5.1 (0.3)		
Triglycerides (mg/dL)	112.5 (53.7)	104.5 (59.5)	107.2 (44.6)		

! Different from 0–9, ^ Different from 10–19, * Different from 20–29, # Different from 30–39, $ Different from 40+, MFI, MetFlex Index™

## 4 Discussion

This study aimed to introduce and quantify a novel marker of the metabolic response to exercise, the MetFlex Index™. Secondarily, this study aimed to characterize associations between the MetFlex Index™ and common health indicators and cardiovascular disease risk factors. This is the first study to characterize the distribution of the MetFlex Index™ across the age spectrum and between males and females. Herein, we showed that MetFlex Index™ scores peak between ages 30–49 years and declines with each subsequent cohort. Aging is known to play a role in the gradual decline in VO_2max_ regardless of fitness levels (Betik and Hepple, 2008) and aging may also reduce lactate clearance capacity (Tzankoff and Norris, 1979). Interestingly, our 18–29 year old cohort presented with a lower average MetFlex Index™ whereas, compared to longitudinal data, we would expect this cohort to be the highest fit (Letnes et al., 2023). It is possible that our sample may be less active than their peers historically given recent trends in sedentary behavior and the earlier onset of metabolic dysfunction.

We also showed that MetFlex Index™ scores were generally higher on average for males than females. This may be expected due to increased lean tissue or percent muscle mass as well as hormonal influences between the sexes (Landen et al., 2023). The study showed significant negative associations between the MetFlex Index™ and measures of body composition relating to fat (visceral fat area, percent body fat, and body fat mass) and significant positive associations relating to muscle (lean body mass, skeletal muscle mass, and percent skeletal muscle mass) suggesting that muscle quantity and quality may play a role in metabolic flexibility. The MetFlex Index™ was also associated with resting vitals. This is to be expected as the MetFlex Index™ is a direct marker of metabolic fitness described through lactate clearance capacity and, therefore, one would expect higher MetFlex Index™ scores to be associated with healthier cardiovascular risk factor profiles. A major finding from Kelley et al. (2018) was a significant inverse association between VO_2max_ and metabolic syndrome and this association was also graded across the range of fitness. Rodriguez et al. (2022) reported that, in addition to VO_2max_, other ventilatory-based variables obtained from CPET were also associated with the presence of metabolic syndrome. The consistent inverse association between fitness and metabolic syndrome persisted regardless of testing modality and method (Kelley et al., 2018). There are currently limited published data describing the association between CRF and cardiometabolic health using lactate-based methods (San-Millán and Brooks, 2018).

Here, the results do not identify significant associations between the MetFlex Index™ and most blood metabolites. This is likely due to the small sample size for our blood metabolite profile data and the sampling distribution bias toward a lower MetFlex Index™ (all participants in the metabolite sample had an MetFlex Index™ < 30). One would expect similar negative associations to what was found for the other cardiometabolic health indicators (Kelley et al., 2018; Rodriguez et al., 2022). Future research relating to metabolic syndrome with larger datasets should continue to analyze potential associations between the MetFlex Index™ and other common blood metabolite profiles including HDL, HOMA-IR, and fasting insulin.

### 4.1 MetFlex Index™, cardiometabolic health, and metabolic flexibility

One would expect that individuals with characteristically reduced metabolic health and metabolic flexibility would also demonstrate a reduced MetFlex Index™. This study showed significant negative associations between the MetFlex Index™ trends and common markers of cardiometabolic health. For example, the metabolic phenotype of an individual with a low MetFlex Index™ is more likely to include high visceral fat, low percent skeletal muscle, high resting heart rate, and a higher blood pressure. Conversely, an individual with a higher MetFlex Index™ is more likely to exhibit low visceral fat, high percent skeletal muscle, low resting heart rate, and a lower blood pressure (Table 4). It may be that the MetFlex Index™ could be considered the first lactate-based scoring system for describing systemic cardiometabolic function and risk stratification given the pattern of inverse associations observed across the range of MetFlex Index™ values observed in this study (Table 4).

Metabolic flexibility is the ability of an organism to adapt fuel availability and use to energy demands. Historical descriptions for changes in bioenergetics and metabolism such as skeletal muscle adaptability (Egan and Zierath, 2013) appear to be moving toward metabolic flexibility and metabolic inflexibility (Mandarino et al., 1996; Kelley et al., 1999). Comprehensive explanations of these concepts and related methodologies have been developed and described in detail elsewhere (Muoio, 2014; Goodpaster and Sparks, 2017; Karwi et al., 2018; Rynders et al., 2018; Smith et al., 2018; Bosch et al., 2020; Harper and Patti, 2020; Tareen et al., 2020; Galgani and Fernández-Verdejo, 2021; Tsilingiris et al., 2021). One method for determining metabolic flexibility has been by calculating changes in the respiratory quotient via indirect calorimetry during an euglycemic-hyperinsulinemic clamp. Observations revealed a reduced ability, or inflexibility, to switch from fat to glucose oxidation in the transition from fasting to insulin with glucose stimulation (Dubé et al., 2014). Individuals with metabolic health impairments including insulin resistance, obesity, and diabetes also had a higher preference for glucose relative to fat as an energy source when fasted further demonstrating metabolic inflexibility. Performing clamp or biopsy studies at scale to describe metabolic flexibility currently appears inefficient and cost prohibitive. Compared to lactate sampling during a GXT such as in this study, clamps and biopsies appear to be feasible as research-based techniques rather than commercially feasible for the following reasons: *i)* access to these methods are typically limited, *ii)* they are relatively invasive with high risk approaches, *iii)* there is generally a longer duration for testing and the time for follow-up analysis and interpretation can be lengthy, *iv)* they require extensive training of skilled personal performing these invasive techniques, and *v)* they are likely cost prohibitive if implemented in the general population.

Lactate has demonstrated utility as a surrogate in characterizing metabolic flexibility alongside traditional methods for measuring substrate oxidation. It may be that the comparison of lactate curves, including threshold(s) identification as frames of reference, can independently provide insight into metabolic flexibility without the need for concurrent methods of sampling including gas exchange, clamps, or biopsies. Moreover, instead of the days to weeks between testing and interpretation with, for example, clamps and biopsies, the interpretation of results is available immediately after the lactate based GXT providing opportunities for same-visit interpretation. It is reasonable that lactate testing, as described here, could be implemented at scale in a variety of wellness and preventative settings, given the low-risk methodology, including fitness centers, physical therapy settings, chiropractic clinics, and hospital-based fitness centers, among others. Although opportunities for scaling lactate testing in various medical-based scenarios are promising given the current associations noted above, additional clinical research with controls for comparison will be necessary to demonstrate diagnostic accuracy, and eventually, therapeutic efficacy.

Lactate thresholds (also referred to as turn-points, transitions, inflections) remain an area of active research and deliberation as currently over twenty methods have been described to date for identification of the first or second lactate thresholds (Jamnick et al., 2020). Lactate thresholds, and the curves they are embedded in relative to the GXT protocol, may provide useful and indirect insights into the current physiological capacity for fuel selection and utilization. The first lactate threshold can be described as the first onset of lactate accumulation relative to a stable, baseline concentration during a steady state activity at a given duration. This threshold is associated with a transition in substrate utilization from peak or maximal fat oxidation as lactate begins to accumulate due to production exceeding clearance capacity or oxidation. The accumulation of lactate may directly or indirectly inhibit, downregulate, or out-compete fat oxidation (Lagarde et al., 2021) and potentially lipolysis. The lower the lactate threshold and clearance capacity relative to power, the lower the fat oxidization and the lower the metabolic flexibility, and *vice versa*. Early lactate accumulation may have an impact on adipose metabolism and *vice versa* (Lagarde et al., 2021) and the early onset of early lactate accumulation may indicate a poor capacity to manage metabolic stress (Poole et al., 2021). The second lactate threshold can be described as the maximal concentration of lactate that can be sustained without progressive accumulation during a steady state activity. This physiological threshold is not of primary interest to these authors and has been described elsewhere, including similar concepts as the respiratory compensation point, the 2^nd^ ventilatory threshold, the maximal metabolic steady state, and critical power.

Evidence suggests that measuring lactate during a GXT can provide insight into the relationship between lactate clearance capacity and metabolic flexibility. In one study, they compared lactate clearance capacity relative to substrate utilization among professional cyclists, moderately fit individuals, and very low fit adults living with metabolic syndrome (San-Millán and Brooks, 2018). Here, lactate clearance and fat oxidation were shown to be strongly negatively correlated, whereas lactate clearance and carbohydrate oxidation were strongly positively correlated (San-Millán and Brooks, 2018). Since lactate (pyruvate) and fat oxidation occur at the level of the mitochondria, observing lactate clearance capacity may afford an indirect observation of mitochondrial capacity.

Mitochondrial structure and function may be involved in metabolic dysfunction considering fat oxidation occurs at the level of the mitochondria and physical activity or inactivity can modulate this process (Jakovljevic et al., 2021; Tsilingiris et al., 2021). This relationship between reduced fat oxidation and increased lactate accumulation (reduced lactate clearance capacity) may provide validity for an additional method of quantifying mitochondrial oxidative capacity and metabolic flexibility as with lactate sampling during a GXT. In a recent pre-publication, San-Millan et al. (2024), building on prior observations (San-Millán and Brooks, 2018), compared sedentary and active individuals during resting and exercising conditions. They observed multiple downregulations across a variety of metabolic assessments (including mitochondrial respiration and bioenergetics) in the sedentary sample in both resting and active states including dysregulated pyruvate and lipid metabolism, consistent with metabolic inflexibility. These findings are also consistent with ventilatory-based data (i.e., VO_2_@VT1) given the first lactate threshold and first ventilatory threshold may share physiological and functional relevance (Gaskill et al., 2023). For example, a gas exchange threshold of 9–11 mL/kg/min has been associated with pre-operative risk and fragility thresholds in community dwelling adults (Older and Levett, 2017). There is currently limited research available exploring risks related to low 1^st^ lactate thresholds (metabolic inflexibility). Here, we demonstrate that the mean predicted submaximal VO_2_ for a given MetFlex Index™ category is lower for lower MetFlex Index™ groups (Table 1) including metabolic-related risks associated with these lower fitness levels (Table 4) which may be relevant for at-risk populations and those living with chronic conditions.

Unlike other CRF assessment methods such as CPET, it may be that the MetFlex Index™, as a function of the first lactate threshold, provides a comprehensive evaluation of peripheral muscle metabolic status and capacity, suggesting that it is a strong indicator of overall fitness. Given that mitochondria are primary location for lactate metabolism and that muscle-based mitochondria are found in greater volume-density within Tyle I oxidative muscle fibers, it may be that the MetFlex Index™ is a useful marker of skeletal muscle oxidative metabolism. Mechanistically, our findings indicate that MFI determined by GXT may be useful for tailoring exercise programs to different populations, potentially optimizing improvements in muscle oxidative metabolism and metabolic flexibility. Future studies might explore associations between the MetFlex Index™ and common peripheral adaptations such as mitochondrial biogenesis or respiratory enzyme activity. Quantifying skeletal muscle metabolism indirectly with a lactate-based biomarker via the MetFlex Index™ adds to the novelty of our approach.

### 4.2 Usefulness of the MetFlex Index™

Using BMI instead of body mass, as in the Watts/kg cycling performance model, affords integration of the long-standing structural biometric relative to physiological capacity, providing additional insight on risk stratification in health and healthcare. This is consistent with individualization of diagnostics, therapeutics, and precision health trends. Here, when defining physiological capacity as the power produced at the first lactate threshold relative to BMI, one can also compare the effectiveness of any intervention claiming to improve oxidative capacity, such as an activity-based program, a nutrition or supplement program, or a pharma-based program. The MetFlex Index™ could be used to monitor a gain or loss in metabolic flexibility over a defined period, regardless of the intervention, or be used to observe the stability, progression, or regression of metabolic flexibility related to the bioenergetics of any disease process or chronic condition of interest. Establishing methods to quantify the relationship between an external load (e.g., power as Watts) to an internal response (e.g., lactate clearance capacity and heart rate) provides further opportunities to develop health outcome-based metrics (Herold et al., 2019). Furthermore, the MetFlex Index™ is observed and defined through submaximal protocols that are effort-independent, affording inclusion and access of a greater population than might complete or repeat a VO_2max_ test.

### 4.3 Safety

No adverse events, infections, or injuries occurred during the 45 months of testing. Submaximal effort testing does not carry the same risk as maximal effort testing (even though this risk is very low as well) and offers relevant physiological-based observations for describing submaximal threshold data.

### 4.4 Strengths and limitations of the current study

Strengths relevant to this study include a large sample size from various community settings (*n* = 826 from several pooled cohorts) in the US and Canada which affords limited generalizability. Another strength is the incorporation of cardiometabolic health indicators associated with metabolic dysfunction.

Limitations of this study include the small sample size biased in lower fitness categories for the blood metabolite profile, a lower percentage of females relative to the general population, and no sampling of race/ethnicity/socioeconomic data. Further, our design was cross sectional which makes it difficult to make any causal inferences or characterize potential changes in MetFlex Index™ over time in response to exercise training or to other interventions like nutrition or pharmaceutical-based. Our selection for cycling as the primary testing modality was influenced primarily by the ability to measure Watts. We acknowledge cycling may influence lactate clearance and production differently than treadmill-based methods.

### 4.5 Future directions

Future directions in this line of research include an exercise training intervention with pre vs post comparison with the testing protocols described herein. With this study design we may better describe how the MetFlex Index™ may be impacted by endurance and/or resistance exercise interventions. Historically, endurance-based exercise like steady state training and interval training have demonstrated effective stimuli (stress) for improving cardiorespiratory fitness over resistance-based methods, although there is growing evidence that resistance-based methods provide more benefits than merely strength. It may be useful to compare various methods of exercise intervention to explore their relative impact on the MetFlex Index™. Further research is needed to determine if potential changes in MetFlex Index™ yield additional benefits to overall health and cardiometabolic health markers. It may be useful to incorporate clinical populations in a study including those living with metabolic syndrome or prediabetes, for example, who may benefit from education and training based on their MetFlex Index™. Additionally, future studies should work to include racial/ethnic/socioeconomic diversity to determine if there are different associations between the MetFlex Index™ and cardiometabolic risk factors or to confirm generalizability. With additional research we may be able to further describe and standardize a submaximal GXT protocol using blood lactate (Chavez-Guevara et al., 2024), and leverage this protocol to develop exercise training recommendations targeting improvement of this metric over time. Moreover, the prospect of real-time sampling of lactate from days to weeks with new wearable technology, such as with continuous lactate monitors and biosensors, provides exciting opportunities to better observe lactate metabolism in various clinical and non-clinical conditions with patterns likely never seen before. The feasibility, or convenience, of large scale market adoption of these testing protocols to characterize the MetFlex Index™ in fitness and healthcare is yet to be determined given the more recent availability and access to these kinds of physiological tests (i.e., lactate, VO_2max_). Current potential barriers to implementation may be in managing a small amount of blood (similar to a glucose meter), insurance coverage or reimbursement models, and education and training around test interpretation.

## 5 Conclusion

The MetFlex Index™ is a novel approach to the characterization of exercise performance across a wide range of ages, and we have demonstrated that there may be clinical utility in its assessment due to the significant associations with this value and several routinely evaluated cardiometabolic risk factors. This approach to fitness evaluation may represent a more inclusive and scalable model for cardiometabolic fitness assessment in the general population including an eventual avenue for individualized exercise prescription for healthy and clinical populations alike. One example of integration in fitness settings would be to train appropriate staff on a lactate testing protocol deployed within a designated fitness testing room with upright and/or recumbent cycles. Here the MetFlex Index™ could be used as a frame of reference for objective fitness goals and responsiveness to a given training method, class, or nutrition program. An example of integration into a clinical setting would be in outpatient physical therapy settings. Here, various staff could provide this method of physiological testing like the fitness setting. The MetFlex Index™ could be used in a variety of ways including pre and post-operative fitness assessment with individualized interventions, return to work or sport rehabilitation guidance, supporting medical weight loss and bariatric programs and outcomes, and supporting Phase 3 cardiopulmonary rehab efforts with individualized programming, virtual monitoring, and accountability.

## Data Availability

The raw data supporting the conclusions of this article will be made available by the authors, without undue reservation.
