# Supplementary figures and images for: Development of the MetFlex Index™: associations between cardiometabolic risk factors and fitness using a novel approach with blood lactate
Source: Front Physiol. 2025 Apr 14;16:1546458. doi: 10.3389/fphys.2025.1546458 (PMC12035540; doi:10.3389/fphys.2025.1546458)

## Slide 1
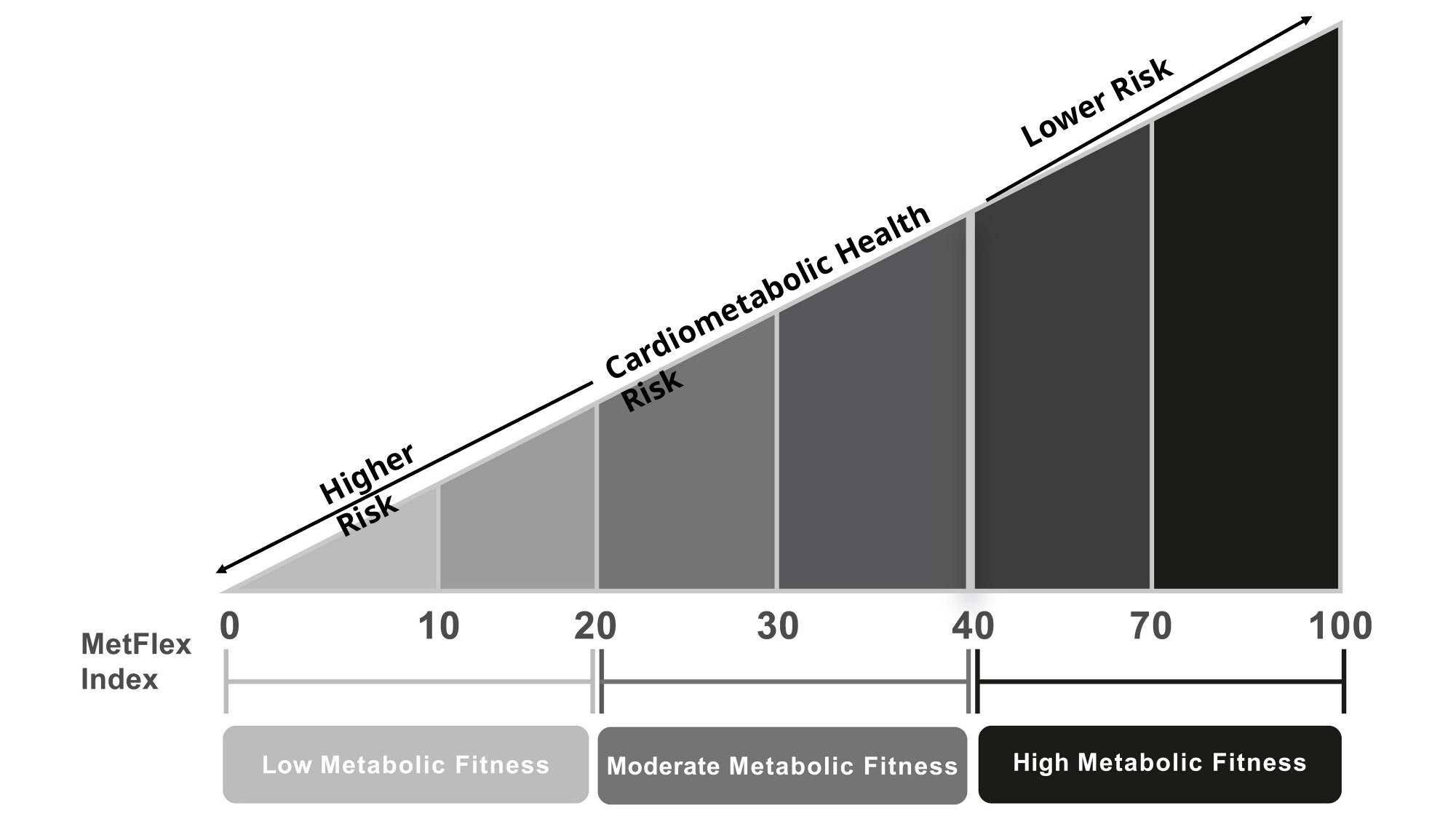

Lower Risk
Cardiometabolic Health Risk
Higher Risk

Supplement: Supplementary file 1 [file Presentation1.pptx]
